# Meconium Microbiome Analysis Identifies Bacteria Correlated with Premature Birth

**DOI:** 10.1371/journal.pone.0090784

**Published:** 2014-03-10

**Authors:** Alexandria N. Ardissone, Diomel M. de la Cruz, Austin G. Davis-Richardson, Kevin T. Rechcigl, Nan Li, Jennifer C. Drew, Roberto Murgas-Torrazza, Renu Sharma, Mark L. Hudak, Eric W. Triplett, Josef Neu

**Affiliations:** 1 Department of Microbiology and Cell Science, Institute of Food and Agricultural Sciences, University of Florida, Gainesville, Florida, United States of America; 2 Department of Pediatrics, University of Florida, Gainesville, Florida, United States of America; 3 Department of Pediatrics, University of Florida, Jacksonville, Florida, United States of America; Vanderbilt University, United States of America

## Abstract

**Background:**

Preterm birth is the second leading cause of death in children under the age of five years worldwide, but the etiology of many cases remains enigmatic. The dogma that the fetus resides in a sterile environment is being challenged by recent findings and the question has arisen whether microbes that colonize the fetus may be related to preterm birth. It has been posited that meconium reflects the in-utero microbial environment. In this study, correlations between fetal intestinal bacteria from meconium and gestational age were examined in order to suggest underlying mechanisms that may contribute to preterm birth.

**Methods:**

Meconium from 52 infants ranging in gestational age from 23 to 41 weeks was collected, the DNA extracted, and 16S rRNA analysis performed. Resulting taxa of microbes were correlated to clinical variables and also compared to previous studies of amniotic fluid and other human microbiome niches.

**Findings:**

Increased detection of bacterial 16S rRNA in meconium of infants of <33 weeks gestational age was observed. Approximately 61·1% of reads sequenced were classified to genera that have been reported in amniotic fluid. Gestational age had the largest influence on microbial community structure (R = 0·161; p = 0·029), while mode of delivery (C-section versus vaginal delivery) had an effect as well (R = 0·100; p = 0·044). *Enterobacter*, *Enterococcus*, *Lactobacillus*, *Photorhabdus*, and *Tannerella,* were negatively correlated with gestational age and have been reported to incite inflammatory responses, suggesting a causative role in premature birth.

**Interpretation:**

This provides the first evidence to support the hypothesis that the fetal intestinal microbiome derived from swallowed amniotic fluid may be involved in the inflammatory response that leads to premature birth.

## Introduction

Preterm birth is the major cause of perinatal morbidity and mortality and is a leading cause of death in children under the age of 5 years old worldwide [1]. The dogma for exclusive postnatal acquisition of microbes is shifting with increasing evidence that the infants' initial inoculum can be provided by maternal transmission before birth [2]. The mechanisms leading to preterm labor are not well understood; an integral role for microbiota in premature birth has been suggested [3], [4]. Microbiological evidence from placental tissue and amniotic fluid samples from preterm deliveries suggests that infection may contribute to approximately 25% of preterm births. Bacterial colonization rates are as high as 79% for birth at 23 weeks of gestation but considerably lower, at 11% at 31 to 34 weeks [4], [5].

Microbes often colonize amniotic fluid from mothers who deliver prematurely (regardless of ruptured or intact membrane), and the quantity of microbial DNA and markers of inflammation correlate inversely with gestational age [6], [7]. Various mechanisms of amniotic colonization have been described including the ascension and translocation of vaginal microbiota [8], [9], as well as via the bloodstream from non-reproductive tissues such as the oral gingiva [10]. The most widely considered paradigm is that once these microorganisms are inside the uterus, they result in the release of proinflammatory cytokines, prostaglandin, and matrix metalloproteases, which lead to cervical ripening, membrane rupture, uterine contractions and preterm birth [11]. It is unclear whether the resulting immune response derives maternally or from the fetus, but studies of blood spots obtained several days postnatally from infants born at different gestational ages suggest a fetal origin of the labor-triggering responses [12].

The site of origin of the fetal inflammatory response is unknown. However, given the higher sensitivity of fetal intestinal tissue to inflammatory stimuli than the sensitivity of mature intestine [13], inflammation-related induction of labor could very likely be derived from the fetal intestine. Fetuses swallow large quantities of amniotic fluid during the late second and third trimesters of pregnancy [14]. This suggests that *in utero* ingestion of microbes present in the amniotic fluid leads to the bacterial colonization of the fetal gut and incites an immune response resulting in the onset of labor. In order to investigate the proximal components of this mechanism, it is essential to evaluate the microbiome of the *in utero* fetal intestinal environment.

Several studies have shown that meconium is not sterile [15]–[17] and contact with microbes is associated with changes in the expression profile of innate immune genes of the fetal intestine [18]. If, indeed, microbes in amniotic fluid have contact with the intestine of the fetus and cause an inflammatory response, then detectable remnants, such as microbial DNA and markers of inflammation would be expected to be present in the meconium of these infants. By analyzing the meconium microbiome from infants of various gestational ages, microbial signatures that correspond with gestational age could indicate organisms that are involved in premature labor. Amniotic fluid is very difficult to obtain routinely at different gestational ages whereas meconium is readily accessible, and may be a reasonable alternative for evaluation of the in-utero microbial environment.

This study aims to determine if there are bacteria from the fetal intestine that correlate with prematurity and also to gain a better understanding of microbial establishment in the human intestine.

## Methods

### Study Patients

Written informed consent was obtained from the infants' parents and investigations were conducted according to the principles expressed in the Declaration of Helsinki. The study including consent procedure was approved by the UF/Shands Institutional Review Boards. IRB# is 386–2008. Meconium was collected from 52 infants at three University of Florida hospitals. The gestational age of subjects ranged between 23 and 41 weeks. Samples were collected from diapers with sterile spatulas, placed in sterile tubes, and frozen at −80°C within 12 hours. ‘Hands on care’ of infants was scheduled every 3 hours, so meconium samples were collected up to 3 hours from the time stool was passed. Additionally, all samples were collected within 48 hours of birth. Metavariables were recorded and analyzed for these samples (Table S1 in File S1).

### DNA Extraction & Amplification

DNA was extracted from approximately 200 mg of meconium sample using FastSpin DNA extraction kit for soil (MP Biomedicals, Santa Ana, CA). DNA was quantified using Nanodrop spectrophotometer (Thermo Scientific, Wilmington, DE). Amplification of the V4 region of 16S rRNA gene was performed using 515F and 806R primers^20^ flanked with Ion Torrent sequencing barcodes and adaptors (Ion Torrent Systems Inc., Guilford, CT). PCR reactions were prepared with 25 µl of GoTaq colorless Master Mix (Promega, Madison, WI), 10 µM of primers, 20 ng of sample DNA adjusted to 50 µl total volume with sterile, nuclease-free water. Samples were then purified using PCR Purification Kit (Qiagen, Hilden, Germany), quantified fluorometrically with QuBit dsDNA High Sensitivity (Invitrogen, Life technologies Inc., Carlsbad, CA), and fragment size was verified on an agarose gel. Neither DNA nor amplification was detected in blank control samples, and any meconium samples in which amplification was not detected were determined to be sterile.

### Sequencing and Data Processing

Multiplexed sequencing reactions were performed on the Ion Torrent PGM platform using four Ion 318™ Chips and the Sequencing 300 bp kit protocol (Ion Torrent Systems Inc., Guilford, CT). An average of 3.9 million reads per chip were generated (average read length of 214 bp). Reads longer than 300 bases were separated by barcode and trimmed using sickle (https://github.com/najoshi/sickle.git); 72.3% of reads were retained after quality filtering. An average of 162,994 (±76,173) reads per sample remained with an average read length of 250 bp (±66 bp). Sequenced reads for non-sterile samples (N = 35) have been deposited on MG-RAST (ID 5358).

Reads were assigned to Operational Taxonomic Units (OTUs) by aligning to the RDP-TaxCollector database with a minimum identity of 95% (USEARCH); 82.6% (±6.6%) of reads were classified in any given sample. OTU contingency tables were created using the taxonomic description at the Domain, Phylum, Class, Order, Family, Genus, and Species levels as denoted by the taxonomic description in the TaxCollector database. Tables were created from USEARCH output and filtered such that each OTU had a minimum of 100 reads in any meconium sample.

Perhaps the largest potential confounder in this work is the possibility for contamination. Sterility of sample collection and processing is supported by the result that 32·7% of all samples had no detectable 16S rRNA and, hence, were assumed to be sterile. The microbes found in meconium samples related closely to those detected by others in amniotic fluid (see below). Furthermore, previous microbial studies of meconium showed that surface community structure of meconium was not distinguishable from the interior suggesting contact with skin is not a primary contributor to the meconium microbiome and hence not an appreciable confounder [19].

### Statistical Analysis

Identification of associations between the presence of 16S rRNA or preterm labor (PTL) and categorical metavariables were assessed using Fisher's exact test (Table S2 in File S1). Confounding variables associated with gestational age were identified (Table S3 in File S1) and further evaluated. Non-metric multidimensional scaling (NMDS) using Bray-Curtis distance and diversity analyses (Shannon-Weaver index and Chao 1 richness) were performed on rarefied count data (sample size of 47,000 reads) using the *phyloseq* [20] package in R. Analysis of similarity (ANOSIM) was used to assess which variables best accounted for microbiome variability. When necessary, gestational age was treated as a categorical variable and grouped by <33 weeks or >33 weeks based on findings that the most serious illnesses associated with preterm birth usually occur in infants born <33 weeks. Spearman correlation analyses and two-sided Mann-Whitney tests were calculated from proportion of total reads using R; all correlations and tests with p<0·05 were considered significant. All analyses and graphics were generated in R version 2.15.0 (R project, Statistical Software): http;//www.R-project. org, and *ggplot2* package, respectively.

## Results

### Gestational age and bacterial colonization of meconium

**Figure 1:**
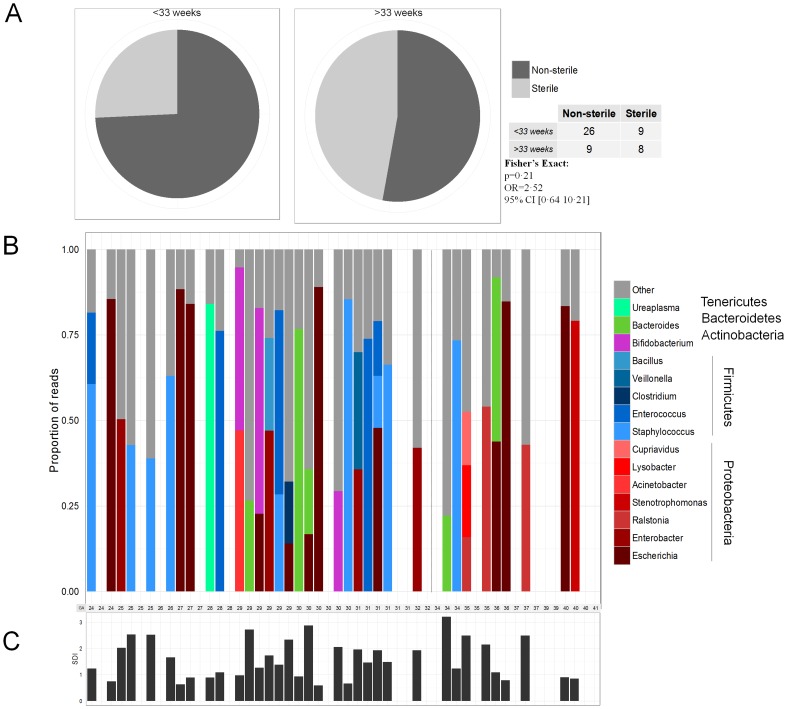
Gestational age and bacterial colonization of meconium. (A) A larger percentage of meconium samples from infants <33 weeks gestational age tend to be colonized (74·3%; N = 35) compared to infants of >33 weeks gestational age (52·9%; N = 17). (B) The bacterial composition of meconium is dominated by few genera; on average the most abundant genera in any given sample comprised 57·3±22·5% of reads; <33 and >33 weeks is displayed by the black, dashed line. (C)Dominant genera contribute to low diversity, measured by Shannon index; this is indicative of a founding population. Furthermore, gestational age was not correlated with Shannon diversity index (Spearman: rho = 0·03, p = 0·85).

Of 52 infants sampled, 16S rRNA was amplified from meconium DNA of 35 subjects (67·3%). A greater percentage of meconium samples were non-sterile in subjects of <33 weeks gestational age (74·3%; N = 35) compared to subjects >33 weeks gestational age (52·9%; N = 17) (Figure 1A), but this was not significant (p = 0·21; Fisher's Exact test). No significant associations were detected between the presence of 16S rRNA or PTL with administration of maternal antibiotics, infant antibiotics, gestational age, mode of delivery, anticipated mode of feeding (breast versus formula), or gender (Table S2 in File S1).

### Dominant genera characterize meconium microbial community – low diversity regardless of gestational age

The microbiome of all non-sterile samples in this study were dominated by a particular genus (Figure 1B), regardless of gestational age. Any one dominant genus accounted for 18%–89% of the microbiome of a given subject. Spikes of Firmicutes-classified genera (shades of blue in Figure 1B) occurred more frequently prior to 33 weeks gestational age (9 of 26 in non-sterile subjects <33 weeks and 1 of 9 in >33 weeks gestational age; Figure 3C, Table 1). No association between several variables and the dominance of particular genera or diversity was detected with the exception of mode of delivery (Table S4 in File S1). The microbiome of infants delivered by cesarean section was more diverse by both Shannon and Chao measurements (p = 0·047 and p = 0·032, respectively; Table S4 in File S1)

**Table 1:** Phyla, family, and genera taxonomy significantly correlated with gestational age (*p<0·05, **p<0·001; Spearman correlation) are reported.

Phylum				Family				Genus			
OTU	<33 weeks	≥33 weeks	p-value	OTU	<33 weeks	≥33 weeks	p-value	OTU	<33 weeks	≥33 weeks	p-value
Firmicutes**	44·5 (±17·6)	17·1 (±14·5)	0·006	Bacillaceae*	2·50 (±2·5)	0·76 (±0·95)	0·031	Bacillus*	2·48 (±2·78)	0·70 (±1·87)	0·032
				Staphylococcaceae*	13·30 (±11·9)	4·71 (±8·89)	0·036	Staphylococcus*	13·28 (±13·21)	4·71 (±17·8)	0·036
				Enterococcaceae**	8·77 (±9·8)	0·43 (±0·53)	0·007	Enterococcus**	8·67 (±11·04)	0·41 (±1·04)	0·007
								Vagococcus*	0·08 (±0·15)	0·01 (±0·04)	0·018
				Lactobacillaceae**	0·83 (±0·81)	0·07 (±0·08)	0·003	Lactobacillus**	0·82 (±0·91)	0·07 (±0·15)	0·003
				Leuconostocaceae*	0·42 (±0·52)	0·02 (±0·02)	0·018	Leuconostoc*	0·42 (±0·60)	0·02 (±0·04)	0·016
				Clostridiaceae*	1·56 (±1·8)	0·35 (±0·50)	0·030	Clostridium*	1·54 (±1·98)	0·33 (±0·96)	0·033
				Peptostreptococcaceae*	1·15 (±2·0)	0·03 (±0·05)	0·033				
				Veillonellaceae*	2·46 (±3·1)	0·39 (±0·59)	0·030	Veillonella*	2·27 (±3·44)	0·30 (±0·99)	0·020
								Negativicoccus*	0·001 (±0·25)	0·0001 (±0·00)	0·011
				Erysipelotrichaceae*	1·26 (±2·5)	0·05 (±0·05)	0·049				
Actinobacteria*	7·6 (±7·6)	1·1 (±1·0)	0·012	Bifidobacteriaceae*	5·52 (±6·7)	0·35 (±05·6)	0·013	Bifidobacterium*	5·47 (±7·65)	0·35 (±1·11)	0·016
Proteobacteria	35·4 (±17·9)	65·6 (±16·5)	0·523	Enterobacteriaceae*	26·32 (±16·9)	15·20 (±15·9)	0·024	Enterobacter**	6·35 (±7·99)	0·06 (±0·14)	0·002
								Citrobacter*	0·19 (±0·23)	0·03 (±0·06)	0·014
								Erwinia*	0·33 (±0·36)	0·03 (±0·07)	0·013
								Klebsiella*	0·60 (±0·64)	0·05 (±0·12)	0·022
								Raoultella*	0·30 (±0·30)	0·21 (±0·44)	0·035
								Pantoea*	0·36 (±0·38)	0·03 (±0·05)	0·011
								Photorhabdus**	0·98 (±1·34)	0·01 (±0·01)	0·002
				Oxalibacteraceae	0·01 (±0·01)	1·08 (±1·23)	0·436	Oxalicibacterium*	0·001 (±0·00)	0·56 (±1·38)	0·030
Bacteroidetes	6·0 (±8·0)	9·6 (±8·4)	0·329	Porphyromonadaceae	0·20 (±0·17)	0·09 (±0·12)	0·087	Tannerella**	0·02 (±0·02)	0·00 (±0·0)	0·004

The mean percent (%) relative abundance and standard deviation for each taxonomic name for meconium samples from infants <33 weeks and >33 weeks gestational age are indicated.

### Meconium microbiome is most suggestive of amniotic fluid origin

**Figure 2:**
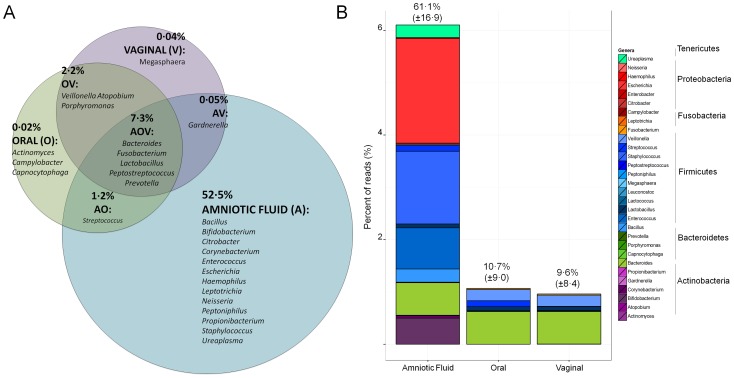
Meconium microbiome is most suggestive of amniotic fluid origin. (A) The average percent relative abundance in meconium samples of this study for genera reported in amniotic fluid, and the oral and vaginal cavities of pregnant women^6,7,27^ are displayed by the Venn diagram which distinguishes unique and shared maternal environments of genera. (B) The potential total mean contribution and standard deviation of any particular maternal locale (amniotic fluid^5,6^, oral^21^, or vaginal^21^), and the phyletic distribution of contributing genera is shown in the stacked bar plot. The color assignment is as follows: Actinobacteria  =  purple; Bacteroidetes  =  green; Firmicutes  =  blue; Fusobacteria  =  orange; Proteobacteria  =  red; Tenericutes  =  aquamarine.

To consider the potential sources contributing to the microbiota found in infant meconium, the meconium microbiome data here were compared to data from various published microbiome studies (Figure 2). Thirty genera found present in studies of amniotic fluid [6], [7], colostrums [21], vaginal canal [22], oral cavity [22], and meconium of full term infants [15] were also identified in meconium samples of this study (Figure S1 in File S1). Genera common between amniotic fluid and the meconium samples of this study accounted for a greater relative abundance (61·1%±16·9) than other sources included in the comparison. Genera identified in the oral and vaginal cavities of pregnant women accounted for 10·7% (±9·0) and 9·6% (±8·4), respectively.

### Confounding variables associated with gestational age

Birth weight, mode of delivery, and infant antibiotic exposures were associated with low gestational age (p<0·05; Table S3 in File S1). No other recorded metadata collected were significantly associated with gestational age.

### Microbiome differences correlated with gestational age

**Figure 3:**
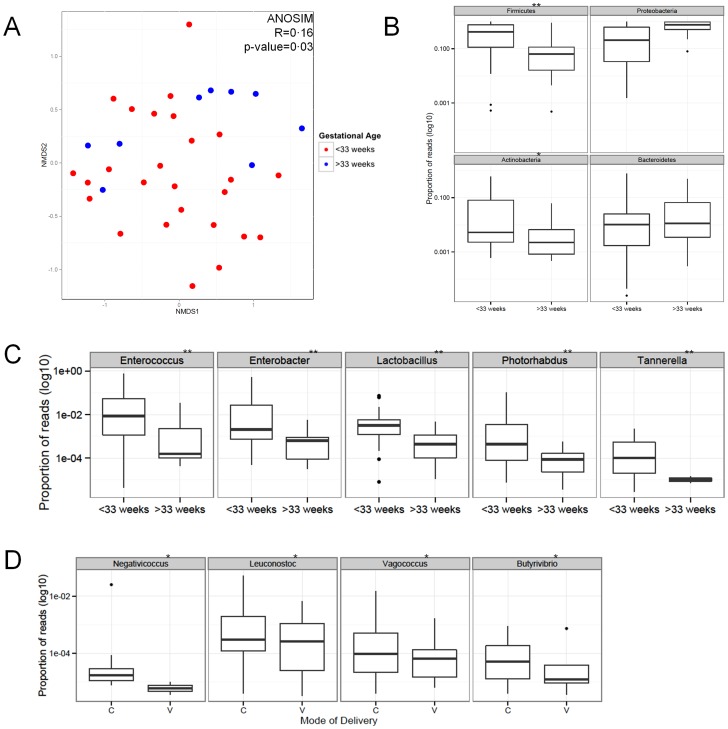
Inflammatory marker S100A12 was correlated with gestational age. (A) Non-metric multidimensional scaling ordination plot depicting the relatedness of the bacterial communities from all meconium samples; communities from >33 week infants (blue) clustered more closely than those from <33 week infants (red). Analysis of similarity (ANOSIM) revealed that gestational age (<33 and >33 weeks) had the largest effect on meconium microbial structure (R = 0·16; p-value = 0·03). (B) Of the four predominant phyla, the relative abundance of Firmicutes and Actinobacteria was correlated with low gestational age (**p<0·01 & *p<0·05, respectively). (C) Genera negatively correlated with gestational age (**p<0·01) are presented. (D) Genera associated with mode of delivery (*p<0·05) were observed, though these differences are not as pronounced as the genera associated with gestational age.

Non-metric multidimensional scaling and analysis of similarity (ANOSIM) using Bray-Curtis distance revealed that microbial communities of <33 weeks gestational age group were different from that of >33 weeks gestational age group (R = 0·16; p = 0·03) (Figure 3A). Additionally, ANOSIM was used to evaluate the effects of collected metavariables on the dispersion of samples in the ordination plot and to further assess the influence of potential confounding factors on the microbiome (Table S5 in File S1). Gestational age had the largest influence on microbial community structure (R = 0·161; p = 0·029), while mode of delivery (MOD) had a lesser but significant effect as well (R = 0·100; p = 0·044).

Proteobacteria and Firmicutes comprised the majority of average reads classified at the phylum level, 35·4% (±17·9%) and 44·5% (±17·6%), respectively in <33 weeks and 65·6% (±16·5%) and 17·1% (±14·5%), respectively in >33 weeks (Table 1). The relative abundances of Firmicutes and Actinobacteria were negatively correlated with gestational age (p-values of 0·006 and 0·02, respectively; Figure 3B, Table 1).

All significant correlations detected were negatively correlated with gestational age with the exception of *Oxalicibacterium* (Table 1). Taxonomic families within the phylum Firmicutes correlated with gestational age included: Bacillaceae, Staphylococcaceae, Enterococcaceae, Lactobacillaceae, Leuconostocaceae, Clostridiaceae, Peptostreptococcaceae, Veillonellaceae, and Erysipelotrichaceae. At the genus level (Fig. 3C), the strongest correlations were attributed to *Enterococcus* (p-value 0·007) and *Lactobacillus* (p-value 0·003) and comprised an average of 8·67% (±11·04%) versus 0·41% (±1·04%) for *Enterococcus* and 0·82% (±0·91%) versus 0·07% (±0·15%) for *Lactobacillus* in <33 weeks and >33 weeks, respectively. Among Actinobacteria, *Bifidobacterium* was significantly correlated (p-value 0·016), comprising 5·47% (±7·65%) compared to 0·35% (±1·11%) of total reads for <33 and >33 weeks, respectively. Proteobacteria genera that were significantly correlated with gestational age were primarily Enterobacteriaceae and those with the strongest correlations included *Enterobacter* (p-value 0·002) and *Photorhabdus* (p-value 0·002) with an average relative abundance of 6·35% (±7·99%) versus 0·06% (±1·14%) for *Enterobacter* and 0·98% (±1·34%) versus 0·01% (±0·01%) for *Photorhabdus* in <33 and >33 weeks, respectively. Found in even lower abundance (mean 0.02% in <33 weeks, and not detected at all in >33 weeks samples), *Tannerella* was identified in these samples and correlated with low gestational age (p-value 0.004).

### Microbiome differences associated with mode of delivery

Based on ANOSIM results (reported above and Figure 3A) and diversity analysis showing that the meconium microbiome is related to with mode of delivery and accounts for differences in diversity (Table S4 in File S1), genera associated with mode of delivery were identified using Mann-Whitney test (Table S6 in File S1). These genera include *Negativococcus* (p = 0·029), *Leuconostoc* (p = 0·036), *Vagococcus* (p = 0·036), and *Butyrivibrio* (p = 0·044) and were found to have a greater relative abundance in cesarean-delivered infants (Figure 3D). All of these genera are Firmicutes and had a low relative abundance.

## Discussion

Recent evidence supporting a paradigm shift away from the dogma that the womb is sterile and that the human infant is thus born sterile is likely to have major applications in human health and disease [2]. A major implication of these findings relates to preterm birth. Accordingly, we hypothesized that swallowed infected amniotic fluid may incite an intestine-derived inflammatory response that induces preterm labor and that the microbes that relate to this response can be non-invasively evaluated in meconium. Here several findings are presented that support this hypothesis.

Differences in microbial colonization are associated with gestational age. The data in this study show that regardless of gestational age, the meconium microbiome is usually characterized by a high relative abundance of a specific genus (Figure 1B), and the microbial communities differ between <33 and >33 weeks. The former supports the notion that initial intestinal colonization is characterized by a founding species which becomes increasingly diverse with age as exposure to several factors affecting the microbiome increases [23]. The difference in community structure can be explained by specific genera that are negatively correlated with gestational age (Table 1). These observations suggest that composition, rather than colonization alone, is a determinant in gestational age.

By comparing the combined relative abundances of genera found in environments possibly contributing to an *in utero* environment (i.e. vaginal, oral) as well as the amniotic fluid, it was found that a larger relative abundance of genera from preterm baby meconium were shared with amniotic fluid samples than from other reported microbial niches (Figure 2). This similarity suggests that meconium is more indicative of amniotic fluid bacterial communities than other niches. Also of interest is that genera attributed to oral and vaginal environments accounted for approximately the same relative abundance in meconium samples (∼10%), which indicates that both potential origins of intrauterine infection and subsequent fetal gastrointestinal colonization can occur and are potentially doing so simultaneously. In addition previous studies have suggested that an altered vaginal flora is implicated in preterm birth, but targeted antibiotic therapy against vaginosis related bacteria has not been beneficial in preventing preterm labor [24]. Another finding that supports the in-utero origin of the meconium microbiome is that the composition of microbes in feces changes markedly from meconium after the first postnatal week [23].

Genera negatively correlated with gestational age (p-value <0·01) and representing >5% in infants <33 weeks of the total bacterial population are *Enterococcus* and *Enterobacter*. Both of these genera have been associated with inciting inflammatory responses in prematurely born infants [25]. Two other genera, *Lactobacillus* and *Photorhabdus*, represented a lower proportion of the bacterial community (∼0·8–1·0%in <33 weeks) but had a greater relative abundance in <33 week samples. It has been shown that lactic acid bacteria induce interleukin-6 (IL-6) production and non-specific immunity [26] In addition, the cytokine IL-6 is higher in amniotic fluid of preterm versus full term births [27]. Although the ability of the increased *Lactobacillus* population observed in this particular study on inciting IL-6 production has not been investigated, taken together, these findings provide a reasonable candidate mechanism. The increased presence of *Lactobacillus* in the meconium of preterm infants suggests that it was also present in amniotic fluid and thus may be involved in triggering events leading to preterm labor. Additionally, *Tannerella* has been implicated as a marker for periodontal infection and preterm birth [28] and the detection of *Tannerella* in samples <33 weeks but not in samples >33 weeks further suggests a role of fetal colonization by oral microbes in preterm birth.

Mode of delivery did have a minor but observable effect on the microbiome (indicated by the ANOSIM results and an increased diversity in cesarean delivered infants); however, this effect is not as great as gestational age. Although, early gestational age was associated with cesarean delivery (p = 0·009; Table S3 in File S1), when mode of delivery was compared to the relative abundance of different genera, the resulting significant genera were different from those correlated with gestational age. This indicates that mode of delivery provides a unique community that is not influenced by gestational age. Others have reported on microbiome changes due to mode of delivery [29], though the differences observed due to mode of delivery are reported from infants that are several days old, not necessarily meconium. While the gut microbiome is undoubtedly, heavily influenced by mode of delivery, we argue that such differences are observable but are not the primary source in meconium and suggest that meconium provide an easily accessible indicator of the in utero microbial environment which is likely to provide insight to the etiology of preterm birth.

In summary, this study generates a novel hypothesis in that it associates microbes found in meconium with prematurity. The known immunoreactivity of the fetal intestine along with the presence of these microbes suggests a fetal intestinal origin for some cases of spontaneous preterm labor. Further identification and characterization of these organisms and their interaction with the host should lead to novel therapies for prevention of many cases of prematurity.

## Supporting Information

File S1 **Supporting tables and figures.**
(PDF)
