# Correction: Meconium Microbiome Analysis Identifies Bacteria Correlated with Premature Birth

**DOI:** 10.1371/journal.pone.0101399

**Published:** 2014-06-26

**Authors:** The PLOS ONE Staff

The title of Figure 3 is incorrect. Please see the corrected Figure 3 here.

**Figure 3:**
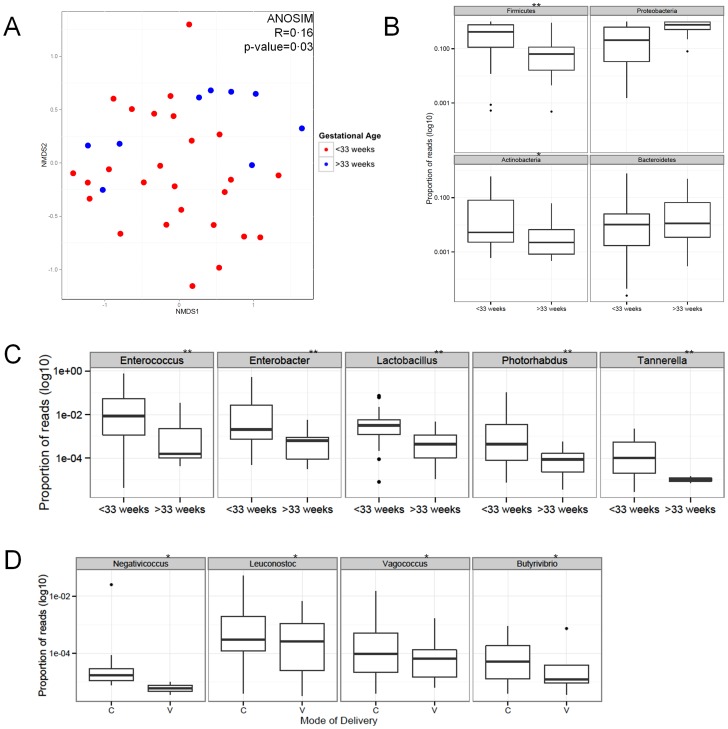
Meconium microbiome correlations with gestational age and mode of delivery. (A) Non-metric multidimensional scaling ordination plot depicting the relatedness of the bacterial communities from all meconium samples; communities from >33 week infants (blue) clustered more closely than those from <33 week infants (red). Analysis of similarity (ANOSIM) revealed that gestational age (<33 and >33 weeks) had the largest effect on meconium microbial structure (R  =  0·16; p-value  =  0·03). (B) Of the four predominant phyla, the relative abundance of Firmicutes and Actinobacteria was correlated with low gestational age (**p<0·01 & *p<0·05, respectively). (C) Genera negatively correlated with gestational age (**p<0·01) are presented. (D) Genera associated with mode of delivery (*p<0·05) were observed, though these differences are not as pronounced as the genera associated with gestational age.
