# Supplementary material for: Meconium Microbiome Analysis Identifies Bacteria Correlated with Premature Birth
Source: PLoS One. 2014 Mar 10;9(3):e90784. doi: 10.1371/journal.pone.0090784 (PMC3948723; doi:10.1371/journal.pone.0090784)
Supplement: File S1 — Supporting tables and figures. (PDF) [file pone.0090784.s001.pdf]

Table S1.

		Frequency	Percent (%)
**16S rRNA**	Non-sterile	35	67·3
	Sterile	17	32·7
**Gestational Age (wk)**	Range<br>Mean (SD)	24-41	31·2<br>(4·7)
**Mode of Delivery**	Cesarean	33	63·5
	Vaginal	19	36·5
**Birth Weight (g)**	Range<br>Mean (SD)	520-4226	1704<br>(905)
**Gender**	F	24	46·2
	M	28	53·8
**Race**	Black	17	32·7
	White	33	63·5
	Other	2	3·8
**Maternal Antibiotics**	Yes	24	46·2
	No	27	51·9
**ROM >18h**	Yes	6	11·5
	No	45	86·5
**Chorioamnionitis**	Yes	8	15·4
	No	40	76·9
**Infant Antibiotics**	Yes	36	69·2
	No	16	30·8
**Intended feeding type**	Formula	7	13·5
	Mix	25	48·1
	Breast Fed	19	36·5
**Insurance**	Medicaid	35	67·3
	Private	14	26·9
	None	3	5·8

Table S2.

		16S rRNA (n=52)		PTL** (n=43)	
		Frequency (%)	P value	Frequency (%)	P value
Gestational age (wk)*	Non-sterile or PTL	30·5*	0·152	28·8*	0·078
	Sterile or no PTL	32·7*		30·7*	
Birth weight (g)*	Non-sterile or PTL	1614*	0·331	1287*	0·118
	Sterile or no PTL	1890*		1682*	
Mode of delivery	Cesarean section (n=33; n=28)	42·3	1	34·9	0·523
	Vaginal (n=19; n=15)	25·0		23·3	
Maternal antibiotics	Yes (n=24; n=21)	28·8	0·546	32·6	0·425
	No (n=28; n=22)	38·5		23·3	
Infant antibiotics	Yes (n=36; n=33)	48·1	0·751	46,5	0·717
	No (n=16; n=10)	19·2		11·6	
Intended feeding type	Formula fed (n=7; n=7)	13·5	0·157	9·3	0·868
	Breast fed (n=19; n=15)	23·1		20·9	
	Mixed (n=25; n=20)	30·8		27·9	
Gender	Female (n=24; n=20)	30·8	1	20·9	0·130
	Male (n=28; n=23)	36·5		37·2	
Insurance	Medicaid (n=35; n=30)	71·4	0·696	56·7	0·593
	Private (n=14; n=10)	57·1		70·0	
	None (n=3; n=3)	66·7		33·3	
ROM (>18h)	Yes (n=6, n=6)	83·3	0·651	50·0	0·685
	No (n=45, n=36)	66·7		80·0	
Chorioamnionitis	Yes (n=8, n=7)	50·0	0·413	40·0	0·418
	No (n=40, n=32)	70·0		50·0	

*Two-sided t-test was calculated for continuous variables, gestational age and birth weight; mean GA or BW is reported in replace of frequency.
**PTL excludes subjects of >37 weeks GA

Table S3.

Metavariable	Test		N		P-value
Birth weight (g)	Spearman correlation	mean	1704g		<0·001
		SD	905g		
Mode of delivery	t-test	C	33		0·009
		V	19		
Infant antibiotic exposures	t-test	Yes	36		0·005
		No	16		
ROM >18h	t-test	Yes	45		0·844
		No	6		
Gender	t-test	F	24		0·658
		M	28		
Maternal antibiotics	t-test	Yes	24		0·995
		No	27		
Feeding	ANOVA	F	7	F-BF	0·393
		BF	19	F-MX	0·600
		MX	25	BF-MX	0·944
Insurance	ANOVA	Medicaid	35	Med-Priv	0·735
		Private	14	Priv-none	0·974
		none	3	Med-none	0·985
Chorioamnionitis	t-test	Yes	8		0·333
		No	40		

Figure S1.

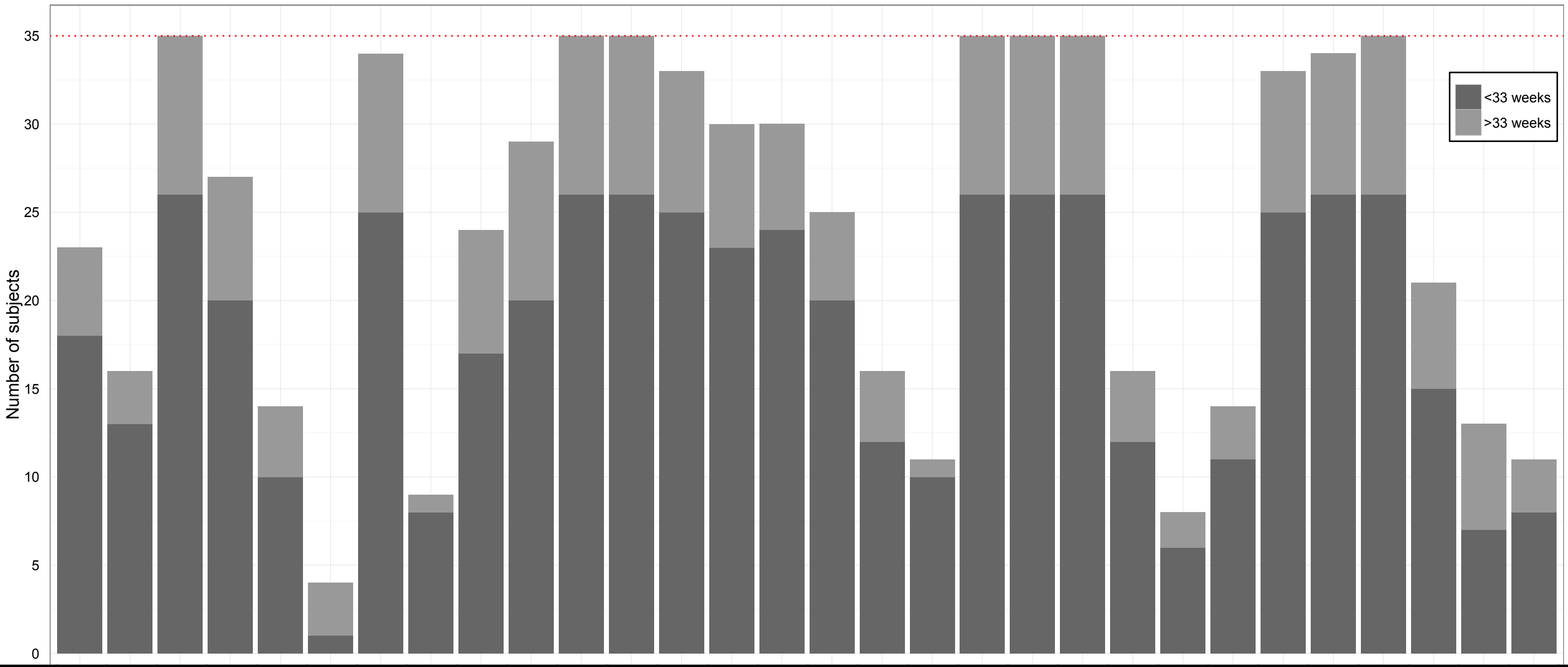

	Actinomyces	Atopobium	Bifidobacterium	Corynebacterium	Gardnerella	Propionibacterium	Bacteroides	Capnocytophaga	Porphyromonas	Prevotella	Bacillus	Enterococcus	Lactobacillus	Lactococcus	Leuconostoc	Megasphaera	Peptoniphilus	Peptostreptococcus	Staphylococcus	Streptococcus	Veillonella	Fusobacterium	Leptotrichia	Campylobacter	Citrobacter	Enterobacter	Escherichia	Haemophilus	Neisseria	Ureaplasma	Mean (SD)	Ref
**AF**			x	x	x	x	x			x	x	x	x				x	x	x	x		x	x		x		x	x	x	x	61·1% (16·9)	6,7
**Colostrum**														x	x				x	x											15·5% (12·6)	20
**Vaginal**		x			x		x		x	x			x			x		x			x	x									9·6% (8·4)	21
**Oral**	x	x					x	x	x	x			x					x		x	x	x		x							10·7% (9·0)	21
**Meconium**												x		x	x				x	x	x				x	x	x				48·6% (17·4)	15

Table S4.

			Shannon Diversity		Chao Diversity	
		Test	Mean (SD)	P value	Mean (SD)	P value
**Gestational age (wk)**		Spearman correlation	1·60 (0·37)	0·848	110·9 (22·4)	0·611
**Birth weight (g)**		Spearman correlation	1·60 (0·37)	0·540	110·9 (22·4)	0·799
**Pre-term Labor (PTL) (n=32)**	PTL (n=18)	t-test	1·62 (0·35)	0·990	124·7 (21·4)	0·076
	no PTL (n=17)		1·61 (0·40)		95·4 (21·7)	
**Mode of delivery**	Cesarean section (n=22)	t-test	1·78 (0·37)	0·047	122·6 (23·1)	0·032
	Vaginal (n=13)		1·29 (0·32)		91·2 (18·0)	
**Maternal antibiotics**	Yes (n=15)	t-test	1·47 (0·38)	0·409	100·3 (17·2)	0·208
	No (n=20)		1·69 (0·36)		118·9 (25·3)	
**Infant antibiotics**	Yes (n=25)	t-test	1·47 (0·40)	0·143	111·0 (24·1)	0·992
	No (n=10)		1·92 (0·34)		110·8 (18·8)	
**Feeding**	Formula-fed (n=7)	ANOVA	1·83 (0·33)	F-MX 1·000	116·1 (18·9)	F-MX 0·970
	Mixed (n=16)		1·83 (0·40)	MX-BF 0·034	120·9 (22·7)	MX-BF 0·283
	Breast-fed (n=12)		1·15 (0·23)	BF-F 0·106	94·6 (23·3)	BF-F 0·571
**Gender**	Female (n=16; n=9)	t-test	1·55 (0·44)	0·721	114·4 (25·8)	0·684
	Male (n=19; n=16)		1·64 (0·31)		107·9 (19·8)	
**Insurance**	Medicaid (n=25)	t-test	1·54 (0·38)	0·779	106·2 (21·3)	0·978
	Private (n=8)		1·75 (0·37)		109·8 (24·1)	
**ROM (>18h)**	Yes (n=5)	t-test	1·30 (0·26)	0·237	112·0 (18·6)	0·948
	No (n=30)		1·65 (0·38)		110·7 (23·3)	
**Chorioamnionitis**	Yes (n=4)	t-test	1·59 (0·46)	0·900	110·8 (23·0)	0·990
	No (n=28)		1·66 (0·37)		110·4 (23·2)	

Table S5.

Variable	R value	p-value
<33 or >33 weeks	0·161	0·029
MOD	0·100	0·044
ROM >18h	0·136	0·143
Location	0·049	0·244
Maternal antibiotics	0·014	0·324
Chorioamnionitis	0·047	0·330
Gender	0·009	0·344
Race	0·005	0·420
Feeding	-0·026	0·663
Insurance	-0·071	0·794
Infant antibiotic exposures	-0·099	0·911

Table S6.

Phylum				Genus			
OTU	C	V	p-value	OTU	C	V	p-value
Firmicutes	31·8 (±35·0)	21·6 (±34·0)	0·310	*Leuconostoc**	0·43 (±1·14)	0·06 (±0·16)	0·036
				*Negativicoccus**	0·09 (±0·47)	0·00 (±0·00)	0·029
				*Vagococcus**	0·08 (±0·28)	0·01 (±0·04)	0·036
				*Butyrivibrio**	0·01 (±0·03)	0·004 (±0·017)	0·044

*p <0.05; Mann-Whitney test
